# Immunohistochemical and molecular study on the protective effect of curcumin against hepatic toxicity induced by paracetamol in Wistar rats

**DOI:** 10.1186/1472-6882-14-457

**Published:** 2014-11-29

**Authors:** Mohamed Mohamed Soliman, Mohamed Abdo Nassan, Tamer Ahmed Ismail

**Affiliations:** Medical Laboratory Department, Faculty of Applied Medical Sciences, Taif University, Turabah, Saudi Arabia; Department of Biochemistry, College of Veterinary Medicine, Faculty of Veterinary Medicine, Benha University, P.O. 13736, Moshtohor, Egypt; Department of Pathology, Faculty of Veterinary Medicine, Zagazig University, Zagazig, Egypt; Department of Physiology, Faculty of Veterinary Medicine, Zagazig University, Zagazig, Egypt

**Keywords:** Curcumin, Hepatic toxicity, Paracetamol, Cytokines expression, MMP-8 immunostaining, Wistar rats

## Abstract

**Background:**

An overdose of paracetamol is a frequent reason for liver and renal toxicity and possible death and curcumin has hepatoprotective properties against liver damage. The exact mechanism of such protection is not clear. Therefore, this study was conducted to examine the molecular levels of the protective effect of curcumin on paracetamol overdose induced hepatic toxicity in rats.

**Methods:**

Male Wistar rats were allocated into 4 groups. Control group, administered corn oil; curcumin group, administered curcumin (400 mg/kg BW daily intra-gastric) dissolved in corn oil; paracetamol group, administered corn oil with a single dose of paracetamol (500 mg/kg BW intra-gastric) and protective group, administered curcumin with a single dose of paracetamol. Curcumin was administered for 7 successive days, while paracetamol was administered at day six of treatment. Blood and liver tissues were collected for biochemical, histopathological, immunohistochemical and molecular examination.

**Results:**

Serum analysis revealed an alteration in parameters of kidney and liver. A decrease in the antioxidant activity of liver was recorded in paracetamol group while curcumin administration restored it. Histopathological findings showed an extensive coagulative necrosis in hepatocytes together with massive neutrophilic and lymphocytic infiltration. Immunostaining of liver matrix metalloproteinase-8 (MMP-8) in paracetamol administered rats showed an increase in MMP-8 expression in the area of coagulative necrosis surrounding the central vein of hepatic lobules. Curcumin administration decreased MMP-8 expression in liver of paracetamol administered rats. Gene expression measurements revealed that paracetamol decreased the expression of antioxidant genes and increased the expression of interleukin-1β (IL-1β), IL-8, tumor necrosis factor-α (TNF-α) and acute phase proteins. Curcumin administration ameliorated paracetamol-induced alterations in genes expression of antioxidant and inflammatory cytokines.

**Conclusion:**

The results clarified the strong protective effect of curcumin on paracetamol induced hepatic toxicity in rats at the immunohistochemical and molecular levels.

## Background

Curcumin (CUR); a member of the ginger family Zingiberaceae; (1,7-bis [4-hydroxy-3-methoxyphenyl] -1,6-heptadiene-3,5-dione) is a hydrophobic polyphenol compound. It is found in the rhizome of the herb *Curcuma longa*, which is commonly known as turmeric [1]. Turmeric is a widely used in therapeutic preparations against anorexia, coryza (rhinitis), herpes zoster, acne, cough, urinary tract diseases, diabetic wounds, hepatic disorder, rheumatism and sinusitis [2]. It is used as a food spice, additive, flavoring, preservative and as coloring agent in foods and textiles [3]. Curcumin has several activities including antioxidant [4], antimicrobial [5], anti-inflammatory [6], antiviral [7], anti-carcinogenic [8] and anti-diabetic [9]. Curcumin has hepatoprotective properties [10, 11] against liver damage in animals induced by carbon tetrachloride [12] and aflatoxin B [13]. Moreover, curcumin has silymarin-like actions [14] and antiapoptotic activity both *in vitro* and *in vivo* to prevent hepatic injury [15].

Paracetamol (PRM); acetaminophen or N-acetyl-p-aminophenol (APAP); is a widely analgesic medication in many countries. An overdose of paracetamol is a frequent reason for liver and renal toxicity and possible death [16]. The exact mechanism of such toxicity is not clear. However, the most studies have focused on PRM effects on antioxidant levels in blood and tissue [15], liver and kidney function [11]. High doses of PRM cause glutathione depletion, apoptosis and cell death [17]. PRM is metabolized in the liver by cytochrome P450 to *N*-acetyl-*p*-benzoquinone imine (NAPQI). NAPQI reacts with glutathione (GSH), therfore overdoses of paracetamol may result in a depletion of hepatocellular GSH [18]. GSH exhaustion will cause NAPQI to binds with cellular proteins leading to mitochondrial dysfunction, oxidative stress, lipid peroxidation, DNA fragmentation, massive hepatocyte necrosis, liver damage and death [19]. The chemicals such as N-acetyl cysteine were used to prevent paracetamol toxicity [20]. Narrow therapeutic window and toxicity together with the adverse effects of N-acetyl cysteine encourage us to search for an alternative safe therapeutic medication to overcome paracetamol overdose and hepatic toxicity.

Matrix metalloproteinases is a family of 23 Zn2^+−^ and Ca2^+−^ dependent endoproteases [21]. These enzymes are very effective in breaking down the major protein components of the extracellular matrix and basement membrane. Matrix metalloproteinase-8 (MMP-8) is a member of metalloproteinases and is a central mediator in acute lethal hepatitis. MMP-8 deficient mice are markedly protected against TNF-α induced lethal hepatitis [22]. Down-expression of MMP-8 is associated with a decrease in mortality of rats with sepsis [23]. MMP-8 regulates expression of tumor necrosis factor-α (TNF-α), interleukin-1β (IL-1β), and other inflammatory cytokines [24].

The liver is a pivotal organ that removes and inactivates toxic substances and drugs to be excreted in urine. Hepatic toxicity is attributed primarily to the changes in oxidative stress and alteration in acute phase proteins [25]. Liver is the first organ to be considered when the effects of environmental pollutants and toxins are investigated. Most of the substances absorbed by the intestine pass first to liver, where toxins and heavy metals are accumulated and inactivated [26]. Therefore, the condition of liver is important for our safety and health and its damage or disease is associated with DNA, protein, and lipid damage [27]. Most of published data focused mainly on serum biochemical alterations induced by curcumin on paracetamol overdose without a precise description about the changes in gene expression occurred during hepatic toxicity. So, the present study was aimed to examine the protective effect of curcumin against hepatic toxicity induced by paracetamol in Wistar rats based on immunohistochemical and molecular studies.

## Methods

### Chemicals and kits

Acetaminophen, ethidium bromide and agarose were purchased from Sigma-Aldrich (St. Louis, MO, USA). The Wistar albino rats were purchased from King Fahd center for Scientific Research, King Abdel-Aziz University, Jeddah, Saudi Arabia. Serologic kits for glutamate pyruvate transaminase (GPT), Glutamate oxalacetate transaminase (GOT), catalase, malondialdehyde (MDA), albumin and urea were purchased from Bio-diagnostic Co., Dokki, Giza, Egypt. The deoxyribonucleic acid (DNA) ladder was purchased from MBI, Fermentas, Thermo Fisher Scientific, USA. Qiazol for RNA extraction and oligo dT primer were purchased from QIAGEN (Valencia, CA, USA). Anti-MMP-8 primary antibody and rat ABC staining system were purchased from Santa Cruz Biotechnology (Santa Cruz, CA, USA).

### Animals, experimental design and sampling

All animal procedures were approved by the Ethical Committee Office of the dean of scientific affairs of Taif University, Saudi Arabia. Forty eight male Sprague Dawley rats, 3 months old, weighing 200–280 g were used for this study. For acclimatization, animals were kept under observation for 7 days before the onset of the experiment. The animals were kept at 12-h light–dark cycle and gained access to food and water ad libitum. Three independent experiments were carried out for each treatment. Rats were randomly divided into 4 groups as follows:

Control group (CTR) served as negative control and received corn oil orally. Curcumin group (CUR) received curcumin dissolved in corn oil orally in a dose of 400 mg/kg BW daily for 7 days. Paracetamol group (PRM) received single intra-gastric dose of paracetamol (500 mg /kg BW intra-gastric) in water, 24 hours before sampling and was receiving corn oil orally for 7 days. Protective group (CUR + PRM) received curcumin dissolved in corn oil (400 mg/kg BW) daily for 7 days and on day six paracetamol (500 mg/kg BW intra-gastric) was administered. The doses of paracetamol and curcumin were determined based on the studies of Zhang et al. [28] and Tarasub et al. [29], respectively. Twenty four hours after administration of tested chemicals, all animals (4 rats per treatment and three independent experiments for each treatment) were sacrificed after anesthetization by diethyl ether inhalation. Blood and tissues were collected from slaughtered rats. Serum was extracted after blood centrifugation for 10 min at 4000 × g. For gene expression, liver tissues were kept in TRIzol® reagent (Life Technologies, USA) at −80°C in deep freezer for ribonucleic acid (RNA) extraction and in 10% neutral buffered formalin (NBF) at room temperature for 24 hours for histopathological and immunohistochemical study.

### Determination of liver antioxidant activity

For catalase and MDA activity measurements, one gram of liver tissues was homogenized in 5ml of cold buffer (50 mM potassium phosphate buffer; PBS, pH 7.4) for catalase and MDA. Cold buffer of catalase activity contains 1 mM EDTA and 1 mL/l Triton X-100. After centrifugation at 4000 × *g* for 15 minutes at 4°C, the supernatant was removed and stored frozen at −80°C until the time of analysis of catalase (U/g tissue) and MDA (nmol/g tissue). The activities of catalase and MDA were determined by ELISA reader (Absorbance Microplate Reader ELx 800TM BioTek®, Seattle, WA, USA). Results were calculated according to the manufacturer’s instructions.

### Liver histopathology

After anesthetization of rats with diethyl ether and sacrifice, the liver was removed and fixed overnight in a 10% NBF solution. Fixed tissues were processed routinely and after washing and preservation in 70% ethanol, dehydration in ascending grades of ethanol, clearing in xylene, paraffin wax embedding, casting and cutting 5 μm sections, they were placed on top of glass slides. The slides were stained with Mayer’s hematoxylin and eosin (H and E) [30]. Tissue slides were visualized using a Wolfe S9-0982 microscope and figures were captured using Canon Power-Shot SX500 IS digital camera.

### Liver immunohistochemical staining of MMP-8

For immunohistochemistry, tissue sections were deparaffinized then treated with 3% H_2_O_2_ for 10 min to inactivate endogenous peroxidases, heated in 10 mM citrate buffer at 121°C for 30 min for antigen retrieval, blocked in 5% normal serum for 20 min, and incubated with a primary polyclonal rabbit anti-rat antibody specific for MMP-8 (1:100 in PBS, Cat # sc-30069; Santa Cruz Biotechnology) overnight at 4°C. After three extensive washes with PBS, sections were incubated with a biotin-conjugated secondary antibody (1:2,000 in PBS; Cat # sc-2040) for 20 min at 32°C. After further incubation with horseradish peroxidase (HRP)-labeled streptavidin, antibody binding was visualized with diaminobenzidine (DAB) and sections were counterstained with hematoxylin for 10 seconds at room temperature based on manufacture instruction. For negative control, primary antibody was replaced with PBS alone. Tissue slides were examined using a Wolfe S9-0982 microscope and images were captured using Canon Power-Shot SX500 IS digital camera. For the expression of MMP-8, five fields per section and 4 sections from 4 different rats per treatment were examined.

### Gene expression analysis

#### RNA extraction

Total RNA was extracted from liver tissue samples (approximately 100 mg per sample) of experimental rats. Liver samples were flash frozen in liquid nitrogen and subsequently stored at −70°C in 1 ml Qiazol (QIAGEN, Valencia, CA, USA). Frozen samples were homogenized using a Polytron 300 D homogenizer (Brinkman Instruments, Westbury, NY, USA). Then, 0.3 ml chloroform was added to the homogenate. The mixtures were shaken for 30 seconds followed by centrifugation at 4°C and 16,400 × *g* for 15 min. The supernatant was transferred to a new set of tubes, and an equal volume of isopropanol was added to the samples, shaken for 15 seconds and centrifuged at 4°C and 16,400 × *g* for 15 min. The RNA pellets were washed with 70% ethanol, briefly dries up, and then dissolved in diethylpyrocarbonate (DEPC) water. RNA concentration and purity were determined spectrophotometrically at 260 nm. The RNA integrity was confirmed in 1.5% denaturated agarose gel stained with ethidium bromide. The ratio of the 260/280 optical density of all RNA samples was 1.7-1.9.

### Complementary deoxyribonucleic acid (cDNA) synthesis

For cDNA synthesis, a mixture of 3 μg total RNA and 0.5 ng oligo dT primer (Qiagen Valencia, CA, USA) in a total volume of 11 μl sterilized DEPC water was incubated in the Bio-Rad T100™ Thermal cycle at 65°C for 10 min for denaturation. Then, 2 μl of 10X RT-buffer, 2 μl of 10 mM dNTPs and 100 U Moloney Murine Leukemia Virus (M-MuLV) Reverse Transcriptase (SibEnzyme. Ak, Novosibirsk, Russia) were added and the total volume was completed up to 20 μl by DEPC water. The mixture was then re-incubated in BIO-RAD thermal cycler at 37°C for one hour, then at 90°C for 10 min to inactivate the enzyme.

### Semi-quantitative PCR analysis

For semi-quantitative RT-PCR analysis, specific primers for examined genes (Table 1) were designed using Oligo-4 computer program and synthesized by Macrogen (Macrogen Company, GAsa-dong, Geumcheon-gu. Korea). PCR was conducted in a final volume of 25 μl consisting of 1 μl cDNA, 1 μl of 10 pM of each primer (forward and reverse), and 12.5 μl PCR master mix (Promega Corporation, Madison, WI, USA), the volume was brought up to 25 μl using sterilized, deionized water. PCR was carried out using Bio-Rad T100™ Thermal Cycle machine with the cycle sequence at 94°C for 5 minutes one cycle, followed by variable cycles (Table 1) each of which consists of denaturation at 94°C for one minute, annealing at the specific temperature corresponding to each primer (Table 1) and extension at 72°C for one minute with an additional final extension at 72°C for 7 minutes. As a reference, expression of glyceraldehyde-3-phosphate dehydrogenase (G3PDH) mRNA was examined (Table 1). PCR products underwent electrophoresis on 1.5% agarose (Bio Basic, Markham, ON, Canada) gel stained with ethidium bromide in TBE (Tris-Borate-EDTA) buffer. PCR products were visualized under UV light and photographed using gel documentation system. The intensities of the bands from four different rats per group and three independent experiments were quantified densitometrically using Image J software version 1.47 (http://imagej.en.softonic.com/).

**Table 1:** **PCR conditions for rat antioxidants, cytokines and acute phase proteins genes**

Gene	Forward primer (5′-3′)	Reverse primer (5′-3′)	PCR cycles and conditions
GST (575 bp)	GCTGGAGTGGAGTTTGAAGAA	GTCCTGACCACGTCAACATAG	35 cycles, 55°C 1 min
GPx (406 bp)	AAGGTGCTGCTCATTGAGAATG	CGTCTGGACCTACCAGGAACTT	40 cycles 57°C 1 min
SOD (410 bp)	AGGATTAACTGAAGGCGAGCAT	TCTACAGTTAGCAGGCCAGCAG	35 cycles, 55°C 1 min
Catalase (652 bp)	GCGAATGGAGAGGCAGTGTAC	GAGTGACGTTGTCTTCATTAGCACTG	35 cycles, 55.5°C 1 min
IL-1β (218 bp)	ATGGCAACCGTACCTGAACCCA	GCTCGAAAATGTCCCAGGAA	30 cycles, 61°C 1 min
TNF-α (256 bp)	CCACCACGCTCTTCTGTCTAC	ACCACCAGTTGGTTGTCTTTG	30 cycles, 58°C 1 min
IL-8 (460 bp)	CTCCAGCCACACTCCAACAGA	CACCCTAACACAAAACAGAT	35 cycles, 56°C 1 min
IL-10 (320bp)	GGAGTGAAGACCAAAGG	TCTCCCAGGGAATTCAAATG	30 cycles, 57°C 1 min
α2 –macroglobulin (325 bp)	GCTCCTGTCTGTTTCCTTAGTT	ATTGGCCTTTCGTGGTTTAG	30 cycles, 56°C 1 min
α1-glycoprotein (230 bp)	GCTTTCCTCCTGACAACGCTG	GGCTTTTTGTTGTTTGCTTCTATTTC	30 cycles, 55°C 1 min
GAPDH (309 bp)	AGATCCACAACGGATACATT	TCCCTCAAGATTGTCAGCAA	25 cycles, 52°C 1 min

### Statistical analysis

Results are shown as means ± standard error of means (SEM). Data were analyzed using analysis of variance (ANOVA) and *post hoc* descriptive tests by SPSS software version 11.5 for Windows (SPSS, IBM, Chicago, IL, USA) with p < 0.05 regarded as statistically significant. Regression analysis was performed using the same software.

## Results

### Renal and hepatic biochemical measurements

Because of the cross association between urea synthesis in liver and its secretion in kidney, the changes in the parameters of kidney and liver after induction of paracetamol toxicity were examined. Paracetamol overdose increased serum levels of GPT, GOT, urea and albumin (Table 2). While, administration of curcumin together with paracetamol inhibited such increase in kidney and liver parameters compared to paracetamol group (Table 2). Curcumin administration alone has no significant effect on examined kidney and liver parameters.

**Table 2:** **Protective effect of curcumin on paracetamol induced changes in serum levels of renal and hepatic parameters in Wistar rats**

	Control	Curcumin	Paracetamol	Curcumin + Paracetamol
**Urea (mg/dl)**	35.6 ± 3.8	35.3 ± 2.3	42.0 ± 1.5*	31.0 ± 1.0^#^
**Albumin (g/dl)**	3.2 ± 0.1	3.1 ± 0.1	4.8 ± 0.4*	3.5 ± 0.1^#^
**GOT (U/L)**	83 ± 2	72.7 ± 9.6	156 ± 9.4*	90.3 ± 3.7^#^
**GPT(U/L)**	58.3 ± 6	64.7 ± 6.01	136 ± 27.9*	98 ± 4.9^#^

Values are means ± standard error (SEM) for 3 independent experiments per each treatment. Values are statistically significant at *p < 0.05 Vs. control and #p < 0.05 Vs. paracetamol group.

### Hepatic antioxidant activity

The results about the protective effect of curcumin on MDA as oxidative stress marker, and catalase as antioxidant enzyme are illustrated in Table 3. The current results revealed that MDA increased significantly (*P* < 0.05) in paracetamol administered rats compared to control group (18.2 ± 0.5 for paracetamol group *vs.* 9.78 ± 1.9 for control). Co-administration of curcumin with paracetamol normalized the increase in MDA activity observed in paracetamol group (12.6 ± 0.4 for paracetamol plus curcumin group *vs.* 18.2 ± 0.5 for paracetamol group). In paracetamol group, catalase activity was decreased significantly compared to control group (21 ± 0.4 for paracetamol *vs.* 33 ± 6.1 for control). For curcumin administered group, there is a significant increase in catalase activity (U/g tissue) (37 ± 1.1 for curcumin *vs.* 9.78 ± 1.9 for control). Administration of curcumin plus paracetamol inhibited significantly the decrease in catalase activity observed in paracetamol group (33.9 ± 2.6 for curcumin plus paracetamol *vs.* 21 ± 0.4 for paracetamol).

**Table 3:** **Protective effect of curcumin on paracetamol induced changes in hepatic antioxidant activity in Wistar rats**

	Control	Curcumin	Paracetamol	Curcumin + Paracetamol
**MDA (nmol/g tissue)**	9.78 ± 1.9	10.7 ± 1.0	18.2 ± 0.5*	12.6 ± 0.4^#^
**Catalase (U/g tissue)**	33 ± 6.1	37 ± 1.1*	21 ± 0.4*	33.9 ± 2.6^#^

Values are means ± standard error (SEM) 3 independent experiments per each treatment. Values are statistically significant at *p < 0.05 Vs. control and #p < 0.05 Vs. paracetamol group.

### Liver histopathology

**Figure 1:**
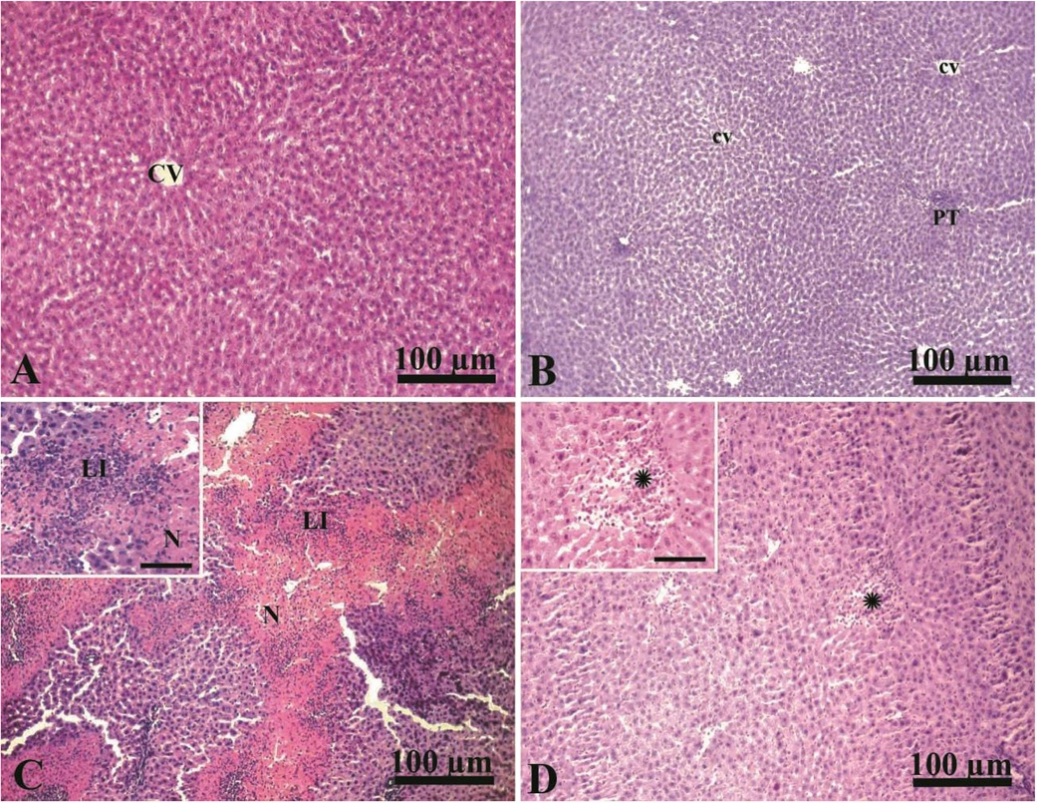
**Photographs of liver histopathology from CTR, CUR, PRM, and CUR + PRM administered rats stained with H & E. A**, liver of CTR group showing normal hepatic architecture with presence of a central vein (CV) surrounded by normal radiating hepatic cords (arrow). **B**, liver of CUR group showing normal hepatic lobules, consisting of a central vein (cv) surrounded by radiating hepatocyte plates with normal portal tracts (PT) surround the classical lobules. **C**, liver of PRM intoxicated group showing extensive coagulative necrosis of hepatocytes (N) together with dense leukocytic infiltration (neutrophils and lymphocytes; LI). **D**, liver of CUR + PRM administered group showing improvement of hepatic toxicity with presence of small degenerated areas (*). Scale bar for all photographs is 100 μm. Inserts are high magnification fields in **C** and **D** with scale bars of 50 μm.

The liver of the control group showed normal hepatic architecture with presence of a central vein surrounded by normal radiating hepatic cords with normal hepatic sinusoids in between (Figure 1A). Liver of curcumin administered rats showed normal hepatic lobules, consisting of a central vein surrounded by radiating hepatocyte plates with normal portal tracts surround the classical lobules (Figure 1B). In contrast, liver of paracetamol intoxicated group showed an extensive coagulative necrosis of hepatocytes together with massive neutrophilic and lymphocytic infiltration (Figure 1C). Interestingly, administration of curcumin together with paracetamol in the protective group showed an improvement of hepatic toxicity with presence of small degenerated area together with normalization in liver architectures (Figure 1D).

### Immunohistochemical staining of MMP-8 in liver

**Figure 2:**
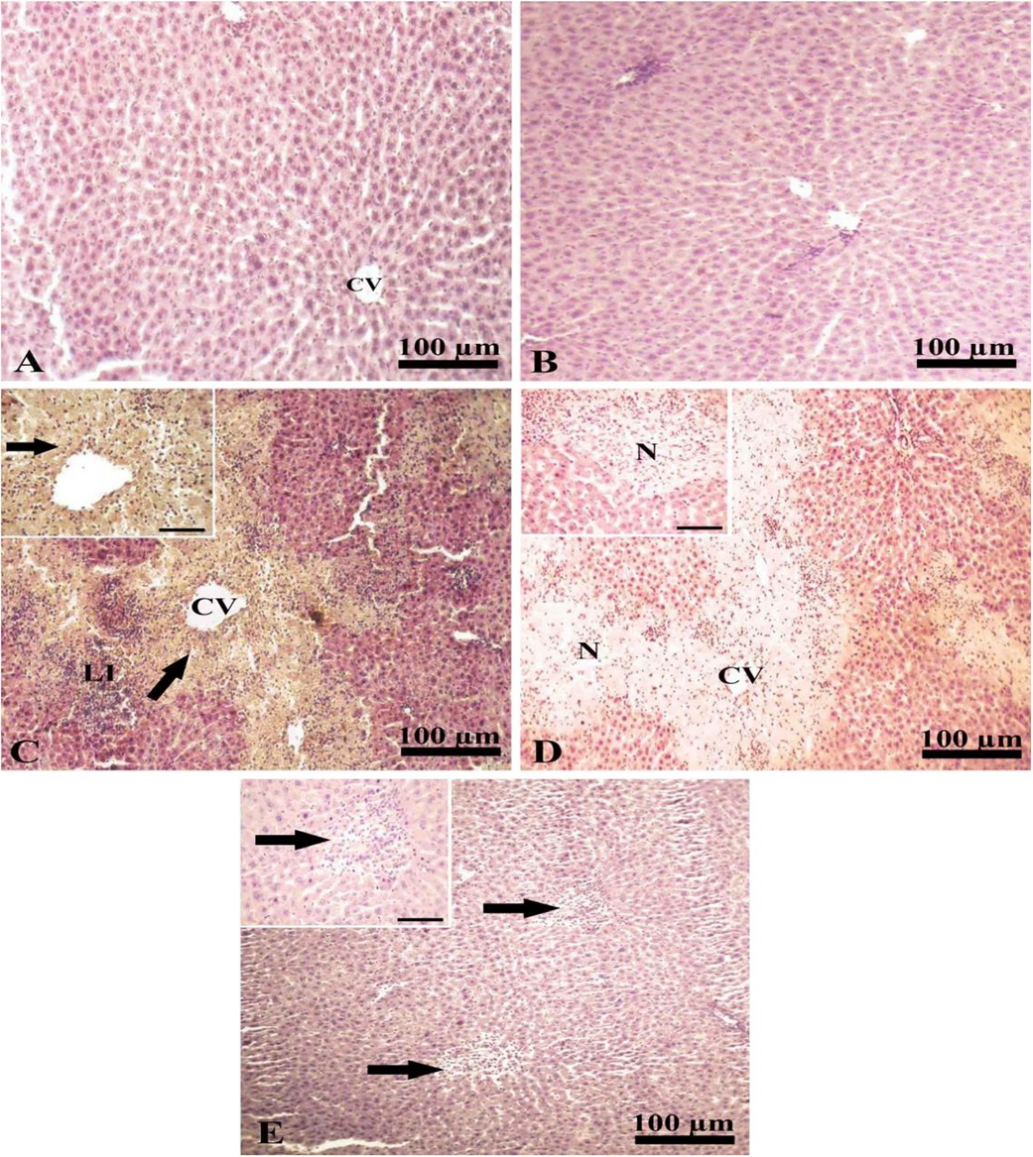
**Immunohistochemical staining of MMP-8 in liver. A** and **B**, liver of CTR and CUR administered groups immunostained showing normal hepatic architecture with presence of a central vein (cv) and normal hepatic cords. **C**, liver of paracetamol intoxicated group showing increased expression of mmp-8 (arrow) in the necrotic area surrounding central vein (cv) together with leukocytic infiltration (neutrophils and lymphocytes; LI). **D**, liver of control negative paracetamol intoxicated group with no expression of mmp-8 in the necrotic area (N) around central vein (CV). **E**, liver of paracetamol intoxicated group treated with curcumin immunostained showing no ­expression of mmp-8 (arrows). Scale bar for photos from A to E is 100 μm. Inserts are high magnification fields in **C**, **D** and **E** with scale bars of 50 μm.

Immuno-stained liver of control and curcumin groups for MMP-8 expression showed normal hepatic architecture (Figure 2A-B). Liver of paracetamol intoxicated group showed an increase in the expression of MMP-8 in the cytoplasm in the area of coagulative necrosis surrounding hepatic central vein (Figure 2C) compared with that of negative control (Figure 2D). The liver of the protective group administered curcumin plus paracetamol showed no expression for MMP-8 (Figure 2E) supporting the protective effect of paracetamol on hepatic toxicity.

### Semi-quantitative RT-PCR analysis of hepatic antioxidant enzymes

**Figure 3:**
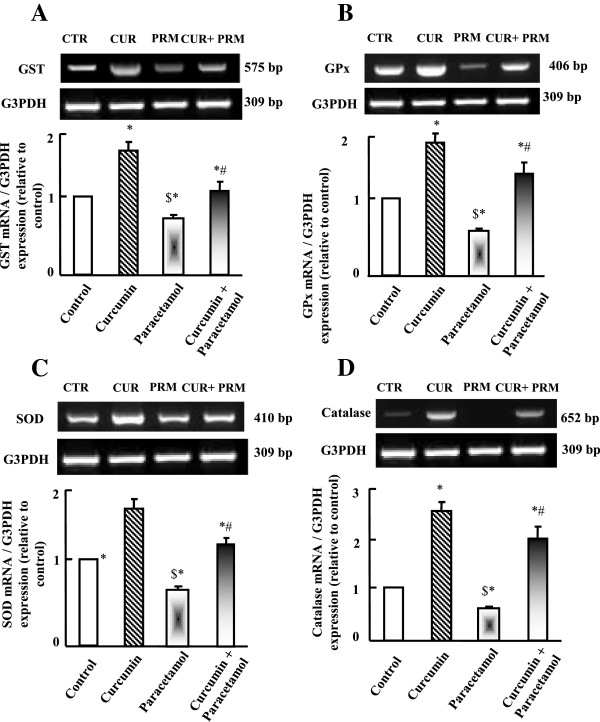
**Semi-quantitative RT-PCR analysis of GST (A), GPx (B), SOD (c) and catalase (D) mRNA expressions and their corresponding G3PDH in liver.** Experimental groups were administered corn oil as a control (CTR), curcumin (CUR), paracetamol (PRM), or curcumin plus paracetamol (CUR+PRM) as described in materials and methods. Values are means ± SEM obtained from 3 independent experiments. P*< 0.05 vs. control group, P$< 0.05 vs. curcumin administered group and P#< 0.05 vs. paracetamol administered group.

RT-PCR analysis for antioxidants expression is illustrated in Figure 3 (A-D). Parallel to tissue catalase activity (Table 3), mRNA expression of glutathione-S-transferase (GST), glutathione peroxidase (GPx), superoxide dismutase (SOD) and catalase was decreased significantly in paracetamol administered group and was increased in curcumin administered group (Figure 3 A-D). Curcumin administration plus paracetamol reversed the decrease in antioxidants expression observed in paracetamol administered groups.

### Semi-quantitative RT-PCR analysis of hepatic cytokines expression

**Figure 4:**
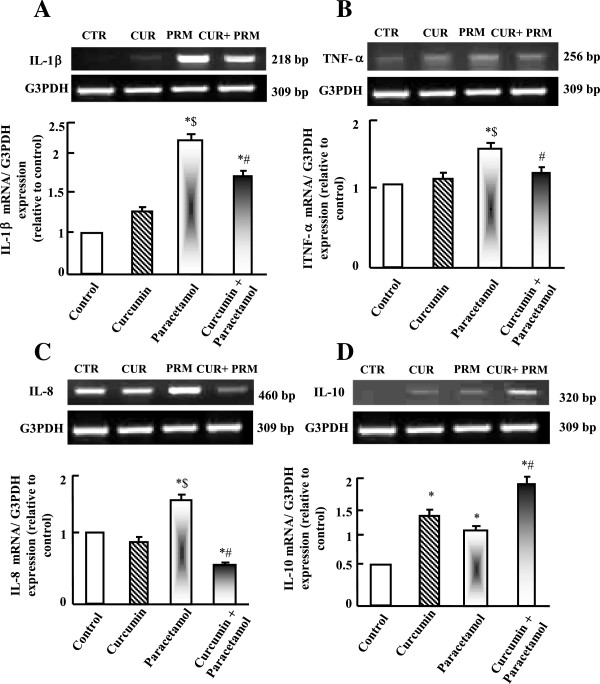
**Semi-quantitative RT-PCR analysis of IL-1β (A), TNF-α (B), IL-8 (C) and IL-10 (D) mRNA expressions and their corresponding G3PDH in liver.** Experimental groups were administered corn oil as a control (CTR), curcumin (CUR), paracetamol (PRM), or curcumin plus paracetamol (CUR+PRM) as described in materials and methods. Values are means ± SEM obtained from 3 independent experiments. P*< 0.05 vs. control group, P$< 0.05 vs. curcumin administered group and P#< 0.05 vs. paracetamol administered group.

Paracetamol up-regulated significantly (p < 0.05) interleukin-1β (IL-1 β) and tumor necrosis factor-alpha (TNF-α) expression compared to control and curcumin administered groups (Figure 4 A-C). Curcumin alone has a minor effect on IL-1β, TNF-α and IL-8 expressions; however, it increased IL-10 expression. Curcumin plus paracetamol administration normalized the increase in IL-1β, IL-8 and TNF-α expressions that are observed in paracetamol administered group. Moreover, curcumin administration plus paracetamol increased the expression of regenerative IL-10 (Figure 4D).

### Semi-quantitative RT-PCR analysis of hepatic acute phase proteins expression

**Figure 5:**
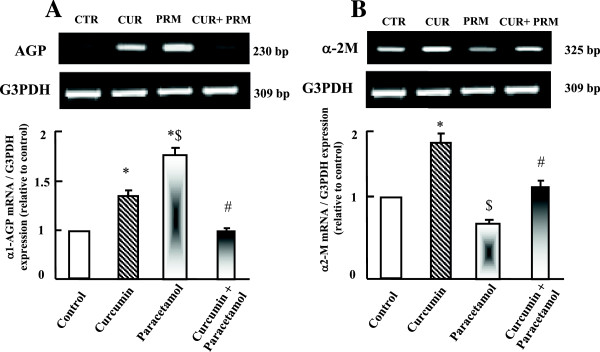
**Semi-quantitative RT-PCR analysis of acute phase proteins AGP (A), and α-2M (B) mRNA expressions and their corresponding G3PDH in liver.** Experimental groups were administered corn oil as a control (CTR), curcumin (CUR), paracetamol (PRM), or curcumin plus paracetamol (CUR+PRM) as described in materials and methods. Values are means ± SEM obtained from 3 independent experiments. P*< 0.05 vs. control group, P$< 0.05 vs. curcumin administered group and P#< 0.05 vs. paracetamol administered group.

To explore the possible involvement of acute phase proteins in curcumin protective effect, the expressions of α1-acid glycoprotein (AGP) and α-2 macroglobulin (α-2M) were examined. AGP expression increased in paracetamol group compared to control and curcumin administered groups (Figure 5A). Curcumin administration plus paracetamol normalized AGP expressions compared to paracetamol, curcumin and control administered groups as shown in Figure 5 (A). Unlike AGP is α-2M mRNA expression, α-2M was down-regulated in paracetamol group compared to control and curcumin administered groups. Curcumin co-administration with paracetamol inhibited the down regulation in α-2M expression compared to paracetamol and control groups (Figure 5B).

## Discussion

This study demonstrated that, curcumin administration attenuated hepatic toxicity induced by paracetamol overdose through the re-impairment of antioxidant capacity of hepatic cells. Curcumin decreased the MMP-8 expression and some cytokines that initiate the inflammatory cascade of the body. In Kheradpezhouh et al. [31] and Yousef et al. [32] studies, they focused on the alterations in serum levels of liver and kidney parameters, but our study focused on the immunohistochemical and molecular attenuation of hepatic toxicity by curcumin.

Alterations in serum levels of hepatic transaminases (GPT and GOT) were used as markers for liver damage and disease. In our study, there was a significant increase in GPT and GOT levels in paracetamol administered rats. Curcumin administration ameliorated both liver and kidney changes confirming the protective role of curcumin against hepatic toxicity induced by paracetamol overdose and that is coincided with results of Li et al. [15]. Moreover, it was reported that curcumin supplementation improved liver histopathology and showed an improvement in hepatic toxicity. MMP-8 plays an important role in progression and regulation of a variety of diseases, inflammatory response, blood pressure and cancer progression [21]. Our results showed an increase in MMP-8 expression during hepatic toxicity and it’s down expression by curcumin in paracetamol group. Curcumin helped in the regeneration process of hepatic cells probably through cytokines expression as reported by our results and that reported by another study [21].

Lipid peroxidation and antioxidant potency (GST, SOD and catalase) of cells were used to assess the degree of hepatic cell stability and integrity [33]. Oxidative damage caused by paracetamol overdose was significantly attenuated by curcumin administration. Therefore, we can postulate that curcumin could protect against free radical mediated oxidative stress by scavenging for free radicals that limit lipid peroxidation and attenuates antioxidants depletion [34]. Curcumin increased mRNA expressions of GST, GPx, SOD, and catalase. Additionally, curcumin attenuates antioxidants depletion and protects liver from paracetamol overdose-induced toxicity. Most of the antioxidants have either a phenolic functional group or a β-diketone group. Curcumin has a variety of functional groups. These functional groups include the β-diketone group, carbon–carbon double bonds, and phenyl rings containing varying amounts of hydroxyl and methoxy substituents [35, 36]. It has been suggested that curcumin was unable to prevent MDA production [37]. Curcumin is bio-transformed to dihydrocurcumin, tetrahydrocurcumin, and hexahydrocurcumin after intestinal absorption. These bio-transformed products are converted to glucuronide conjugates, which are more polar and have better absorption than curcumin. Therefore, the pharmacological actions of curcumin are mostly due to curcumin’s hydrosoluble derivatives [38].

The phenolic and methoxy groups on the benzene rings of curcumin are important structural features that contribute to curcumin’s antioxidant properties [39] and ability to reduce the amount of free radicals [40]. To confirm the antioxidant and anti-inflammatory activity of curcumin during hepatic toxicity, we examined the expression of antioxidants and acute phase cytokines. The antioxidant gene expression and secretion increased in curcumin administered rats compared to control group. Curcumin administration down-regulated the increase of IL-1β, TNF-α and IL-8 expressions in paracetamol administered group [4]. IL-1β, TNF-α, and IL-6 are the major inducers of acute phase response [41]. They act as hepatotrophic factors as evidenced by circulating levels of TNF-α and IL-1β that are increased in rats with liver damage [42]. Curcumin modulates the inflammatory response by down-regulating the activity of cyclooxygenase-2, inducible nitrous oxide synthase, TNF-α, IL-1β, IL-6 and IL-8 secretion [43]. The results confirmed that curcumin decreased mRNA expression of IL-1β, TNF-α and IL-8 that are increased in liver of paracetamol administered group. In the current results curcumin regulated IL-8 expression in a way to initiate chemoattractant mechanism and consequently ameliorate inflammation. The inhibitory effect of curcumin on inflammatory cytokines expression is attributed to the reduction of the Iκ/NF-κB signaling pathway [10]. Moreover, curcumin co-administration with paracetamol increased expression of IL-10, which is a known regenerative cytokine [44]. IL-10 is produced mainly by monocytes with pleiotropic actions [45]. IL-10 down regulates T helper 1 cytokines expression, inhibits IL-1 and IL-6 production [44], configures the development of the immune response and decrease pro-inflammatory cytokine expression [46]. Therefore, the increase in IL-10 expression is a mean to control the degree of toxicity induced by paracetamol and to counteract the increase in expression of IL-1β and TNF-α.

The changes in plasma protein levels of acute phase reaction proteins cause modifications in the way of drug action, distribution in tissues, degradation and elimination [47]. One of the most interesting proteins of acute phase reaction is AGP. AGP is the principal basic protein that binds to drugs with significant clinical implications to control the inflammation cascade in the body [47, 48]. For example, AGP is involved in some pharmacokinetics of some drugs such as drug-drug interactions to induce clinical consequences to reduce the degree of toxicity and inflammation [47]. Therefore the increase in AGP after paracetamol administration is reflex to indicate the degree of toxicity. Curcumin administration attenuated the upregulation of AGP expression to control the inflammation degree occurred in liver.

Alpha 2-macroglobulin is a large plasma protein produced mainly from liver and locally by macrophage, fibroblast and adrenocortical cells [49]. As known, α-2M inhibits fibrinolysis (inhibits plasmin synthesis) and acts as a protein carrier for numerous growth factors and cytokines among which is IL-1β [50]. α-2M secretion is decreased during acute liver inflammation [50]. In current study, α-2M expression is upregulated after curcumin administration and downregulated after paracetamol administration. Previous curcumin administration protected liver cells from paracetamol toxicity to counteract the biohazards induced by paracetamol through normalization of α-2M expression.

## Conclusions

In summary, the current study showed that curcumin attenuates hepatic toxicity induced by paracetamol. The protective effect of curcumin occurred through the upregulation in antioxidants gene expression and down-regulation in oxidative stress markers. Moreover, curcumin regulated MMP-8 and various cytokines expressions. Further *in vitro* studies are needed to outline the signaling pathways involved in curcumin actions during hepatic toxicity.

## Abbreviations

AGP: Alpha1-acid glycoprotein
APAP: N-acetyl-p-aminophenol
cDNA: Complementary deoxyribonucleic acid
CUR: Curcumin
DAB: Diaminobenzidine
DEPC: Diethylpyrocarbonate
DNA: Deoxyribonucleic acid
EDTA: Ethylenediamine tetra acetic acid
G3PDH: Glyceraldhyde-3-phosphate dehydrogenase
GSH: Glutathione
GPT: Glutamate pyruvate transaminase
GOT: Glutamate oxalacetate transaminase
GPx: Glutathione peroxidase
GST: Glutathione-S-transferase
H and E: Hematoxylin and eosin
HRP: Horseradish peroxidase
IL-1: Interleukin 1
MMP-8: Matrix metalloproteinase-8
MDA: Malondialdehyde
M-Mul V: Moloney Murine Leukemia Virus
NAPQI: N-acetyl*- p*-benzoquinone imine
NBF: Neutral buffered formalin
PRM: Paracetamol
PBS: Phosphate buffer saline
RNA: Ribonucleic acid
RT-PCR: Reverse transcription polymerase chain reaction
SEM: Standard error of the mean
SOD: Superoxide dismutase
TNF-α: Tumor necrosis factor alpha
TBE: Tris-borate-EDTA
α-2M: α-2macroglobulin.
